# Association of oxidative stress and inflammatory metabolites with Alzheimer’s disease cerebrospinal fluid biomarkers in mild cognitive impairment

**DOI:** 10.1186/s13195-024-01542-4

**Published:** 2024-07-30

**Authors:** Shahzad Ahmad, Wei Yang, Adelina Orellana, Lutz Frölich, Itziar de Rojas, Amanda Cano, Mercè Boada, Isabel Hernández, Lucrezia Hausner, Amy C. Harms, Margot H. M. Bakker, Alfredo Cabrera-Socorro, Najaf Amin, Alfredo Ramírez, Agustín Ruiz, Cornelia M. Van Duijn, Thomas Hankemeier

**Affiliations:** 1 Department of Epidemiology, Erasmus Medical Centre, Rotterdam, The Netherlands; 2 Metabolomics and Analytics Center, Leiden Academic Centre for Drug Research, Leiden University, Einsteinweg 55, 2333 CC Leiden, The Netherlands; 3 Oxford-GSK Institute of Molecular and Computational Medicine (IMCM), Centre for Human Genetics, Nuffield Department of Medicine, University of Oxford, Oxford, UK; 4 Ace Alzheimer Center Barcelona – Universitat Internacional de Catalunya, Barcelona, Spain; 5 Networking Research Center On Neurodegenerative Diseases (CIBERNED), Instituto de Salud Carlos III, Madrid, Spain; 6 Department of Geriatric Psychiatry, Central Institute of Mental Health, Medical Faculty Mannheim, University of Heidelberg, 68159 Mannheim, Germany; 7 Discovery Research, AbbVie Deutschland GmbH & Co. KG, 67061 KnollstrasseLudwigshafen, Germany; 8 Janssen Pharmaceutical NV, Turnhoutseweg 30, 2340 Beerse, Belgium; 9 Nuffield Department of Population Health, University of Oxford, Big Data Institute, Li Ka Shing Centre for Health Information and Discovery, Old Road Campus, , Headington-Oxford, OX3 7FZ UK; 10 Department for Neurodegenerative Diseases and Geriatric Psychiatry, University of Bonn, Bonn, Germany; 11 Division of Neurogenetics and Molecular Psychiatry, Department of Psychiatry and Psychotherapy, Medical Faculty, University of Cologne, Cologne, Germany; 12 German Center for Neurodegenerative Diseases (DZNE), Bonn, Venusberg-Campus 1, 53127 Bonn, Germany; 13 Excellence Cluster On Cellular Stress Responses in Aging-Associated Diseases (CECAD), University of Cologne, Joseph-Stelzmann-Strasse 26, 50931 Cologne, Germany; 14 Department of Psychiatry and Glenn Biggs Institute for Alzheimer’s and Neurodegenerative Diseases, San Antonio, TX USA

**Keywords:** Alzheimer’s disease, Mild cognitive impairment, *APOE*, Cerebrospinal fluid, Oxidative stress, Isoprostane, Prostaglandin

## Abstract

**Background:**

Isoprostanes and prostaglandins are biomarkers for oxidative stress and inflammation. Their role in Alzheimer's disease (AD) pathophysiology is yet unknown. In the current study, we aim to identify the association of isoprostanes and prostaglandins with the Amyloid, Tau, Neurodegeneration (ATN) biomarkers (Aβ-42, p-tau, and t-tau) of AD pathophysiology in mild cognitive impairment (MCI) subjects.

**Methods:**

Targeted metabolomics profiling was performed using liquid chromatography-mass spectrometry (LCMS) in 147 paired plasma-CSF samples from the Ace Alzheimer Center Barcelona and 58 CSF samples of MCI patients from the Mannheim/Heidelberg cohort. Linear regression was used to evaluate the association of metabolites with CSF levels of ATN biomarkers in the overall sample and stratified by Aβ-42 pathology and *APOE* genotype. We further evaluated the role of metabolites in MCI to AD dementia progression.

**Results:**

Increased CSF levels of PGF2α, 8,12-iso-iPF2α VI, and 5-iPF2α VI were significantly associated (False discovery rate (FDR) < 0.05) with higher p-tau levels. Additionally, 8,12-iso-iPF2α VI was associated with increased total tau levels in CSF. In MCI due to AD, PGF2α was associated with both p-tau and total tau, whereases 8,12-iso-iPF2α VI was specifically associated with p-tau levels. In *APOE* stratified analysis, association of PGF2α with p-tau and t-tau was observed in only *APOE* ε4 carriers while 5-iPF2α VI showed association with both p-tau and t-tau in *APOE* ε33 carriers. CSF levels of 8,12- iso-iPF2α VI showed association with p-tau and t-tau in *APOE* ε33/*APOE* ε4 carriers and with t-tau in *APOE* ε3 carriers. None of the metabolites showed evidence of association with MCI to AD progression.

**Conclusions:**

Oxidative stress (8,12-iso-iPF2α VI) and inflammatory (PGF2α) biomarkers are correlated with biomarkers of AD pathology during the prodromal stage of AD and relation of PGF2α with tau pathology markers may be influenced by *APOE* genotype.

**Supplementary Information:**

The online version contains supplementary material available at 10.1186/s13195-024-01542-4.

## Background

Oxidative stress represents a series of adaptive responses as a result of the insufficiency of the antioxidant system counteracting the oxidant system [1]. Characterized by the excessive production of free radicals like reactive oxygen species and reactive nitrogen species, oxidative stress results in cellular injury, which has been involved in various disorders including neurodegenerative diseases [2, 3] such as Alzheimer’s disease (AD) [4–6]. Besides the tissue damage, oxidative stress may also influence blood–brain integrity which may also activate neuroinflammation [7], an early-stage process in AD pathophysiology [8, 9].

A large number of studies have shown elevated cerebrospinal fluid (CSF) and plasma levels of isoprostanes and prostaglandins in AD [5, 10–16], but their relation with established Amyloid, Tau, Neurodegeneration (ATN biomarkers: amyloid-beta 42 [Aβ-42], phosphorylated-tau [p-tau], and total-tau [t-tau]) [17] is not yet studied during the prodromal phase of AD or linked to the progression from mild cognitive impairment (MCI) to AD dementia. To study the oxidative stress and inflammatory pathways during the prodromal phase of AD, i.e., MCI, we profiled a set of isoprostanes and prostaglandins in both CSF and plasma. Isoprostanes are prostaglandin-like metabolites produced by free radical-mediated phospholipid peroxidation [18] and are established biomarkers of oxidative stress [19]. Together with their isomeric prostaglandins, pro-inflammatory metabolites [20], they reflect oxidative stress combined with inflammatory status [21]. The apolipoprotein E (*APOE*) genotype plays a substantial role in oxidative stress and inflammation. The *APOE* gene is polymorphic and consists of three alleles, *ε4, ε3*, and *ε2*, of which *ε3* is the most common allele in populations. The *ε2* allele of *APOE* is considered protective, while the *APOE* ε4 allele is a major genetic risk factor for AD [22], with carriers exhibiting increased susceptibility to oxidative damage in the brain. Such individuals often exhibit compromised antioxidant defenses, resulting in elevated levels of oxidative stress that contribute to neurodegeneration [23, 24]. Consequently, examining the role of *APOE* in the relationship between oxidative stress and inflammatory markers and AD pathology may be relevant.

Our study aims to determine whether oxidative stress and inflammation-related metabolites in the prostaglandin and isoprostane pathway in CSF and plasma, are associated with Aβ-42, p-tau and t-tau levels in CSF during the prodromal phase of AD. We further studied the influence of *APOE* on the association of metabolites with ATN biomarkers and the progression from MCI to AD dementia.

## Methods

### Study populations

Study participants included in the analyses came from two cohorts of the Alzheimer's Disease Apolipoprotein Pathology for Treatment Elucidation and Development (ADAPTED) consortium, including Barcelona-based memory clinic Ace Alzheimer Center Barcelona (147 CSF-plasma paired samples) and Heidelberg/Mannheim memory clinic (58 CSF samples). Demographic information of the full data set is provided in Supplementary Table 1. Both cohorts had obtained their approvals from their respective medical ethical committees, and informed consents are available from all participants which permit the use of phenotype and biomarker information for research purposes. Due to missing information on BMI and lipid-lowering medication use, MCI patients with complete information on age at blood collection, sex, body mass index (BMI), lipid-lowering medication use, as well as AD biomarkers in CSF (i.e., Aβ-42, p-tau, and t-tau) were selected for both studies (ACE cohort = 142, Heidelberg/Mannheim cohort = 40).

### Ace Alzheimer Center Barcelona cohort

Patient recruitment and assessment was carried out at the Memory Disorders Unit from Ace Alzheimer Center Barcelona (ACE), Spain between 2016 and 2017 [25]. The diagnosis was assigned for each patient by consensus among neurologists, neuropsychologists, and social workers at a case conference. All the MCI patients fulfilled the MCI Petersen’s diagnostic criteria [26, 27] including subjective memory complaints, decline from normal general cognition, preserved performance in activities of daily living, absence of dementia, and a measurable impairment in one or more cognitive functions, with or without a deficit in other cognitive domains (amnestic MCI: single domain or amnestic MCI: multiple domains). The cut-off scores for impairment were based on age and different levels of education. Specific cutoffs for all tests included in the comprehensive neuropsychological battery (NBACE) are detailed elsewhere [28]. Any individual scoring below the established cutoffs [28] in any test was considered to have MCI. In the subsequent follow-up of MCI patients, dementia diagnosis was performed based on the Diagnostic and Statistical Manual of Mental Disorders (DSM)-V criteria [29]. The cognitive deficits within the dementia group were classified according to the 2011 National Institute of Aging- Alzheimer´s Association (NIA-AA) [30] for Alzheimer´s disease; the National Institute of Neurological Disorder and Stroke and Association Internationale pour la Recherche et l’Enseignement in Neurosciences criteria (NINDS-AIREN) [31] for vascular dementia, Frontotemporal Dementia [32], and for Lewy body dementia [33]. Paired CSF and plasma samples were collected from fasted patients using clinically recommended approaches. Lumbar puncture (LP) was used for CSF collection from the patient’s intervertebral space of L3-L4 according to standard recommendations [34] and the procedure was performed by experienced neurologists under local anesthesia (1% mepivacaine) of the patient in a sitting position. Two tubes (10-ml polypropylene tube, Sarstedt ref 62,610,018) of CSF were obtained passively of which, one tube for basic biochemistry analysis including glucose, total proteins, proteinogram, and cell type and cell number. The second CSF tube was aliquoted into polypropylene tubes (Sarstedt ref 72,694,007) after being centrifuged (2000xg 10 min at 4°C) and finally stored at -80°C. This was performed within 2 h after CSF collection. For AD biomarker analysis on the sample collection day, an aliquot was thawed at room temperature and vortexed for 5–10 s followed by CSF Aβ1-42, t-tau, and p-tau level determination using commercially available enzyme-linked immunosorbent assays, namely Innotest Aβ1-42, Innotest hTAU Ag and Innotest PHOSPHO-TAU (181P) (Innotest, Fujirebio Europe) [34–36].

*APOE* genotyping was performed in the ACE cohort. The patient’s whole blood was obtained for DNA extraction using DNA Chemagen technology (Perkin Elmer). Then TaqMan probes analysis (Real-Time PCR QuantStudio3, Thermofisher) was applied to characterize the *APOE* genotype of the patient.

### Heidelberg/Mannheim memory clinic sample

Heidelberg/Mannheim memory clinic cohort included 58 MCI patients between 2012 and 2016 at the Memory Clinic of the Central Institute of Mental Health (Mannheim, Germany). Patients were recruited by detailed medical history, physical and neuropsychiatric examination, and standard serum laboratory assessment excluding subjects with neuropsychiatric or general medical causes of impaired cognition. Therefore, all MCI patients met the MCI Petersen’s diagnostic criteria [26, 27], including subjective memory complaints, normal general cognition, only minimally impaired performance in instrumental activities of daily living, absence of dementia, and a measurable impairment in one or more cognitive domains. Cognitive impairment was defined as performance below 1.2 standard deviation in one or more cognitive domains in standard neuropsychological test battery [37] (test battery of the Consortium to Establish a Registry for Alzheimer Disease (CERAD) [38] plus the Wechsler memory scale – logical memory (WMS) immediate and delayed recall [39], and the trail making test A (TMT-A) and B (TMT-B) [40]. CSF collected by lumbar puncture was used for biomarker assessment and for amyloid determination, and the results of the individual patient were discussed at a case conference attended by geriatric psychiatrists and neuropsychologists. The diagnosis of MCI due to AD or prodromal AD [41] was assigned by consensus. CSF samples were collected and aliquoted for storage at -80°C. Determination of Aβ1-42, p-tau, and t-tau were performed based on standardized protocols in the Neurochemistry Laboratory at the Department of Neurology, University Medical School, Göttingen. CSF levels of p-tau, total-tau and CSF levels of Aβ1-42 were both quantitatively determined using a commercially available ELISA kit [INNOTEST® PHOSPHO-TAU(181P) Innogenetics], INNOTEST® hTAU AG and a commercially available ELISA kit [INNOTEST®β- AMYLOID (1–42) Innogenetics] from Fujirebio respectively. Aβ-40 was measured with ELISA-Kits from IBL. Illumina GSA1.0 Shared Custom Content bead array was applied for *APOE* genotyping. *APOE* genotype determination was performed using GenomeStudio 2.0 software and data were exported in PLINK format.

### Metabolomics profiling

All CSF and plasma samples of both cohorts were analyzed using an ultra-high-performance liquid chromatography tandem mass spectrometry (UHPLC–MS/MS) based approach profiling oxidative stress and inflammatory metabolites including isoprostanes and prostaglandins [42, 43].

Samples were stored at -80°C, thawed on ice, and randomized prior to analysis. The sample volume of CSF aliquot and plasma aliquot was 350 µL and 150 µL respectively. The remains were pooled and used for quality control (QC) samples. CSF samples were dried under the vacuum, spiked with deuterated internal standards (ISTDs) and antioxidant (BHT:EDTA 1:1, 0.2 mg/mL) and then extracted with a mixture of 1-butanol:ethyl acetate (1:1, v/v). After the supernatant was collected and dried, samples were reconstituted using a mixture of methanol: water (70:30, v/v). Plasma samples were prepared with the same ISTDs and antioxidant with extra acidifying buffer of 0.2M citric acid and 0.1M disodium hydrogen phosphate (pH 4.5). Then liquid–liquid extraction was performed with a mixture of 1-butanol:ethyl acetate (1:1, v/v) and samples were vortexed followed by centrifugation and collection of the upper organic phase for evaporation. Dried samples were reconstituted with a mixture of ice-cold methanol: water (70:30, v/v). All reconstituted samples were measured using a Shimadzu LCMS-8050 system (Shimadzu, Japan).

For both plasma and CSF samples, LC–MS analyses were performed with high pH run and low pH run using two aliquots from each reconstituted sample. The high pH run targets 24 lysophosphatidic acid species of which results were published elsewhere [42]. The low pH run targets 16 isoprostanes and their isomeric prostanoids as well as some nitro-free fatty acids. For low pH run, samples were measured using an Acquity BEH C18 column (2.1 × 50 mm, 1.7 µm, Waters) with a tertiary mobile phase system of (A) water with 0.1% acetic acid, (B) 75% acetonitrile with 25% methanol and 0.1% acetic acid, and (C) 100% isopropanol. Dynamic multiple reaction monitoring (dMRM) mode with fast polarity switching was selected for MS acquisition.

QC samples and blank samples were injected together with study samples to ensure data quality. Metabolites showing a relative standard deviation (RSD) no more than 30% on corrected peak areas in QC samples were used as a criterion for metabolite export and further analysis. After QC correction, 9 and 2 metabolites in CSF and plasma, respectively, were used for further data analysis (Supplementary Table 2). We detected two isoprostanes in both CSF and plasma including 8-iso-PGF2α and 8,12-iso-iPF2α VI. Metabolites exclusively detected in CSF samples included three prostaglandins and four isoprostanes. The inverse rank transformation was performed to normalize the distribution of metabolites in both cohorts.

### Association of AD biomarkers with metabolites in CSF and plasma

We performed linear regression to assess the association of Aβ-42, p-tau, and t-tau with the isoprostanes and prostaglandins profiled in paired CSF and plasma samples from the ACE cohort and only CSF samples from the Heidelberg-Mannheim memory clinic. Levels of Aβ-42, p-tau, and t-tau in CSF were used as an outcome variable in the regression model, and the analyses were adjusted for age, sex, body mass index (BMI), and lipid-lowering medications. Information about Aβ-40 and Aβ-42/ Aβ-40 ratio was only available in the Mannheim/Heidelberg cohort, therefore association analysis of Aβ-40 and ratio was only conducted in one cohort. The inverse rank transformation was applied to normalize the distribution of both CSF AD biomarkers (Aβ-42, p-tau, and t-tau) and metabolite levels in CSF and plasma. A meta-analysis of regression analysis results of the two cohorts was performed using METAL software [44] using the inverse-variance fixed-effect model. Meta-analysis results of association were also corrected for multiple testing separately for each AD biomarker using false discovery rate (FDR) by Benjamini and Hochberg method [45] and findings with *FDR* < 0.05 were considered significant in overall analysis. All analyses were performed in R version 4.2 (https://www.r-project.org/).

### Sensitivity analysis

To evaluate the relevance of observed associations between metabolites and AD biomarkers with AD brain pathology, we repeated the association analysis in stratifying MCI patients into Aβ positive and Aβ negative categories. In the ACE cohort, Aβ positive was defined as Aβ-42 < 676 pg/ml and in the participants from Mannheim Heidelberg cohort, Aβ positive was define as MCI participants with Aβ-42 ≤ 550 pg/ml or an Aβ-42 / Aβ-40 ratio < 0.55.

### Comparison of CSF metabolite levels between ATN categories

To further corroborate on the association results of linear regression between metabolites and AD biomarkers, we categorized the patients with MCI based on three AD biomarker categories: Aβ-42 (A ±), p-tau (T ±) and total tau (N ±). We grouped MCI into four categories based on ATN biomarkers to investigate the relationship of metabolites with AD pathology including A-T-N-, A + T-N-, A + T + N- and A + T + N + . We compared the mean values of metabolites between different ATN categories using two tailed t test. Multiple testing correction was performed using *FDR* < 0.05 based on Benjamin and Hochberg method [45]. In the ACE cohort, A + was defined as Aβ-42 < 676 pg/ml, T + as p-tau > 58 pg/ml, and N + as t-tau levels > 367 pg/ml [36]. In the participants from Mannheim Heidelberg cohort, A + was defined as participants with Aβ-42 ≤ 550 pg/ml or an Aβ-42/Aβ-40 ratio < 0.55, T + as p-tau ≥ 61 pg/ml, and N + as total tau ≥ 450 pg/ml. ATN comparison analyses were performed on the full dataset since we have not adjusted the analysis for covariates.

### *APOE* stratified regression analysis

To identify *APOE* specific associations of metabolites with AD biomarkers, *APOE* stratified analysis was performed in both participating cohorts based on three *APOE* strata including *APOE* ε4 (ε4ε4/ ε3ε4/ ε2ε4), *APOE* ε3 (ε3ε3), and *APOE* ε2 (ε2ε2/ε2ε3). In the stratified analysis, subjects with *APOE* ε2ε4 genotype were pooled with patients having *APOE* ε4ε4/ ε3ε4 genotypes based on their similar risk profiles as reported in an earlier study [46]. The *APOE* stratified analyses were adjusted for age, sex, body mass index (BMI), and lipid-lowering medications. *APOE* stratified analysis results were reported as a combined meta-analysis of both datasets (ACE CSF cohort and Heidelberg/Mannheim cohort) included in the current study. Due to the smaller number of *APOE* ε2 carriers in these two datasets, a combined regression analysis was performed, aggregating all *APOE* ε2 carriers from two cohorts. The combined analysis for *APOE* ε2 stratum was additionally adjusted for cohort information in the tested model. The multiple testing correction was performed using *FDR* < 0.05 based on Benjamin and Hochberg method [45].

### Association of *APOE* with metabolite levels

To evaluate the association of *APOE* genotype with metabolites measured in CSF, we also performed the association of metabolites (as outcome) with *APOE* (Predictor) using linear regression analysis. In this analysis, we tested three *APOE* binomial categories: *APOE* ε4 versus *APOE* ε3 carriers, *APOE* ε2 versus *APOE* ε3 carriers and *APOE* ε2 versus *APOE* ε4 carriers using linear regression adjusted for the age, sex, BMI, and lipid-lowering medications. Analysis results were reported for each cohort as well as their combined meta-analysis. We also performed adjusted analysis of covariance (ANCOVA) test to compare three categories of *APOE* (*APOE* ε2 versus *APOE* ε3 versus *APOE* ε4).

### MCI to AD dementia progression analysis

Follow-up information was available for 138 out of 142 MCI patients of the ACE cohort, of which 43 MCI progressed into AD dementia (31%) while 95 MCI did not progress to AD dementia. The criteria for dementia diagnosis are detailed above in the Ace Alzheimer Center Barcelona cohort description. We analyzed the association of metabolites with MCI to AD dementia progression using cox proportional hazard model adjusted for age at blood collection, sex, BMI, and lipid-lowering medication used (Model 1). In the second model, we also adjusted the analyses for *APOE* status. To identify the association of metabolites with MCI to AD progression in different *APOE* carriers, we performed a *APOE* stratified analysis in *APOE* ε4 and *APOE* ε3 carriers.

## Results

The general characteristics of the ACE (discovery) and Heidelberg/Mannheim (replication) cohorts with full information of the covariates used in the analysis are presented in Table 1. The patients of the ACE cohort (Mean age = 71.95, SD = 7.74) were on average 3 years older (*P* = 0.043) compared to the replication cohort (Mean = 68.85, SD = 8.51). The proportion of women was similar (ACE cohort: 52.11%, Mannheim/Heidelberg cohort: 55%) between the two cohorts (*P* = 0.886), and the percentage of patients treated with lipid-lowering medication in the ACE cohort (44.37%) was 1.6 times higher compared to the Heidelberg/Mannheim i.e., 27.5% (*P* = 0.083). Aβ-42, p-tau, and t-tau in CSF levels were not significantly different between the two cohorts. Information about the comorbidities was available for Mannheim/Heidelberg cohort which is provided in the Supplementary Table 1.

**Table 1:** Population description

	**ACE cohort**	**Heidelberg/Mannheim cohort**	***P*-value of difference**
MCI patients (N)	142	40	
Metabolomics profiling tissue	CSF and Plasma	CSF	
Age (SD) blood collection, years	71.95(7.74)	68.85(8.51)	0.043
Female (%)	74(52.11%)	22(55%)	0.886
Body Mass index (SD)	26.47(3.75)	25.86(3.62)	0.354
Lipid-lowering medication user (%)	63(44.37%)	11(27.5%)	0.083
Amyloid-beta 42 in pg/mL (SD)	791.59 (337.36)	690.85 (394.14)	0.151
P-Tau in pg/mL (SD)	71.37 (37.31)	63.17 (29.97)	0.153
Total tau in pg/mL (SD)	425.87 (288.88)	380.95 (326.98)	0.435
*APOE* genotype N (%)			
*APOE* ε4 (ε4ε4/ε3ε4/ε2ε4)	50 (35.21%)	18 (45%)	0.344
*APOE* ε3ε3	81 (57.04%)	18 (45%)	0.242
*APOE* ε2ε2/ε2ε3	11 (7.75%)	4 (10%)	0.895
Amyloid positive	68 (47.89%)	22 (55%)	0.445
ATN categories			
A-T-N-	43 (30.28%)	12 (30%)	0.873
A + T-N-	19 (13.38%)	11 (27.5%)	0.094
A + T + N-	9 (6.34%)	4 (10%)	0.757
A + T + N +	40 (28.17%)	7 (17.5%)	0.140

*Abbreviations*: *MCI* mild cognitive impairment, *SD* Standard deviation, *CSF* Cerebrospinal fluid, *APOE* apolipoprotein E gene

### Association of metabolites with ATN biomarkers in meta-analysis

**Fig. 1:**
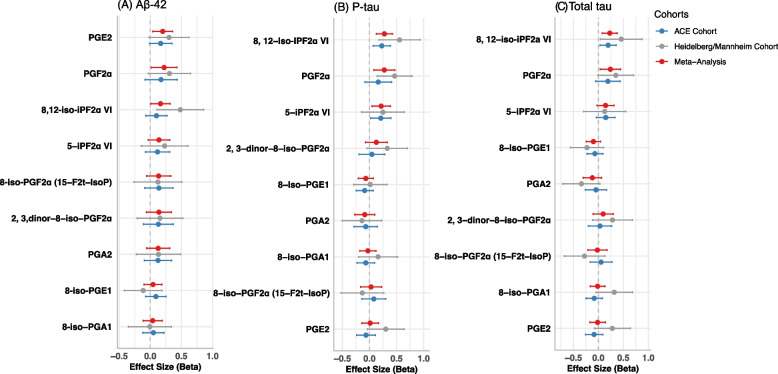
Forest plot of the association of cerebrospinal fluid (CSF) metabolite levels with amyloid beta 42 (**A**), phosphorylated tau (**B**), total tau levels (**C**). Metabolites within each plot are ordered based on their meta-analysis p-values

Results of association analysis of metabolites measured in CSF with ATN biomarker (Aβ-42, p-tau, and t-tau) levels in CSF are provided in Fig. 1 and Supplementary Table 3. In the meta-analysis of association of metabolites with Aβ-42 (Fig. 1A and Supplementary Table 3), none of the metabolites studied were significantly associated when adjusting for multiple testing. Three metabolites showed evidence of association with Aβ-42 including PGE2 (β = 0.20, *P* = 1.33 × 10^–2^), PGF2α (β = 0.223, *P* = 3.36 × 10^–2^) and 8,12-iso-iPF2α VI (β = 0.165, *P* = 3.74 × 10^–2^) that was consistent across cohorts, but all had *FDR* > 0.05. In Mannheim/Heidelberg cohort, four metabolites PGE2 (β = 0.432, *P* = 3.34 × 10^–3^), PGF2α (β = 0.452, *P* = 1.76 × 10^–3^), 8,12-iso-iPF2α VI (β = 0.478, *P* = 5.97 × 10^–3^), and 5-iPF2α VI (β = 0.391, *P* = 2.01 × 10^–2^) showed significant association with CSF Aβ-40 levels (Supplementary Table 4). In the association of metabolites with p-tau (Fig. 1B and Supplementary Table 3), increased levels of 8,12-iso-iPF2α VI (β = 0.275, *P* = 2.0 × 10^–4^), 5-iPF2α VI (β = 0.216, *P* = 1.30 × 10^–2^) and PGF2α (β = 0.273, *P* = 6.0 × 10^–3^) showed significant association (*FDR* < 0.05) with p-tau levels in CSF. While the meta-analysis showed a significant association of PGF2α and 5-iPF2α VI, individual cohort analyses did not show significant associations in one cohort (*P* < 0.05). The regression coefficients for 5-iPF2α VI were similar across two cohorts. However, PGF2α showed a threefold increase in regression coefficient in the smaller Heidelberg/Mannheim sample compared to the larger cohort, suggesting considerable heterogeneity (*I*^*2*^ = 53.4, *P* = 0.143). The isoprostane 8,12-iso-iPF2α VI (β = 0.228, *P* = 3.0 × 10^–3^) was also significantly associated with t-tau levels at *FDR* < 0.05, while PGF2α (β = 0.241, P = 0.020) showed relationship with CSF t-tau levels, mainly driven by the smaller cohort Heidelberg/Mannheim. The 5-iPF2α VI did not show significant association with total tau levels (Supplementary Table 3). The forest plot (Fig. 1) of overall meta-analysis has shown that the association of PGF2α, 5-iPF2α VI, and 8,12-iso-iPF2α VI was similar across the ATN markers but was strongest and most *FDR* significant for p-tau.

### Sensitivity analysis in Aβ positive and Aβ negative MCI participants

In the Aβ positive-MCI participants (Supplementary Table 5), CSF levels of PGF2α (β = 0.470, *P* = 1.10 × 10^–3^) and 8, 12-iso-iPF2α VI (β = 0.326, *P* = 1.57 × 10^–3^) remained significantly associated with p-tau, while PGF2α was associated with total-tau (β = 0.429, *P* = 4.07 × 10^–3^) after multiple testing correction (*FDR* < 0.05). In the current meta-analysis of p-tau, we also did not observe a significant difference of regression coefficients of PGF2α between two cohorts (*I*^*2*^ = 0, *P* = 0.571) as we did in original meta-analysis. We did not identify the association of PGF2α and 8, 12-iso-iPF2α VI with p-tau/t-tau in the Aβ negative-MCI participants (Supplementary Table 6).

### Association of plasma levels metabolites with AD biomarkers

In plasma-based metabolic measurements, only two metabolites (8,12-iso-iPF2α VI, 8-iso-PGF2α) were detected in more than 60 percent of participants. We observed a significant correlation of 8-iso-PGF2α levels between plasma and CSF (correlation coefficient = 0.31, *P* = 1.8 × 10^–4^), no correlation was observed for 8,12-iso-iPF2α VI (correlation coefficient = 0.076, *P* = 0.37) (Supplementary Fig. 1). The observed significant correlation of 8-iso-PGF2α levels in plasma and CSF in ACE cohort, supports its similar association results both in plasma (Supplementary Table 7, Aβ-42: β = 0.040, *P* = 0.652; P-tau: β = 0.020, *P* = 0.818; t-tau: β = 0.026, *P* = 0.761) and CSF (Supplementary Table 3, Aβ-42: β = 0.139, *P* = 0.230; P-tau: β = 0.080, *P* = 0.482; t-tau: β = 0.054, *P* = 0.630). Plasma levels of 8,12-iso-iPF2α VI did not show association with Aβ-42 (β = 0.180, *P* = 0.107), p-tau (β = -0.115, *P* = 0.296) and t-tau (β = -0.144, *P* = 0.183) (Supplementary Table 7).

### CSF metabolite levels between ATN categories

**Fig. 2:**
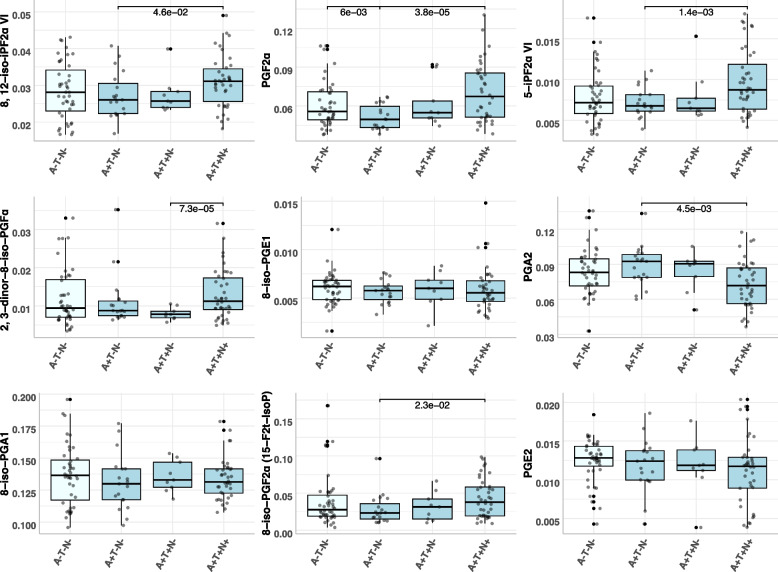
Boxplots illustrating the concentration differences of metabolites across ATN groups. Each p-value is calculated from a two-tailed t-test assessing the mean differences between groups. P-values are displayed only when they are less than 0.05

In our analysis of metabolite levels across ATN categories within the ACE cohort (Fig. 2 and Supplementary Table 8), we identified significantly increased levels of PGF2α (*P* = 3.8 × 10^–5^) and 5-iPF2α VI (*P* = 1.4 × 10^–3^) in A + T + N + compared to A + T-N-. A similar pattern was observed for 8,12-iso-iPF2α VI (*P* = 4.6 × 10^–2^) and 8-iso-iPF2α (*P* = 2.3 × 10^–2^); however, these associations were no longer significant after adjusting for multiple comparisons. The CSF levels of 2,3-dinor-8-iso-PGF2α were also significantly higher in A + T + N + patients compared to A + T + N- patients (*P* = 7.32 × 10^–5^), which might indicate its high correlation with PGF2α levels (Supplementary Fig. 2). Furthermore, we observed significantly lower levels of PGA2 in A + T + N + compared to A + T-N- (*P* = 4.5 × 10^–3^), and decreased PGF2α levels in A + T-N- compared to A-T-N- in MCI patients (*P* = 6 × 10^–3^).

**Fig. 3:**
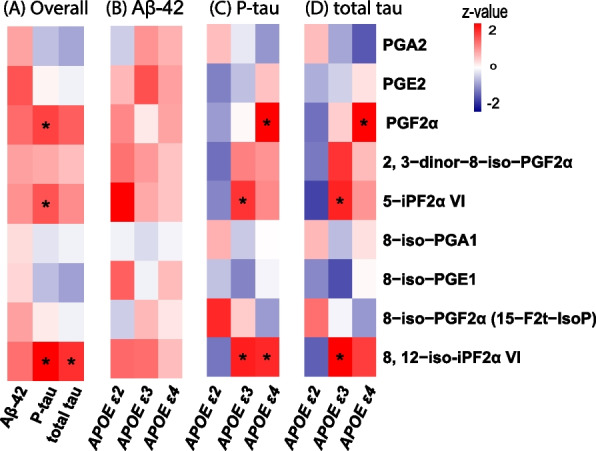
Heatmap of meta-analysis results from regression analyses of oxidative stress metabolites with Aβ-42, p-tau, and t-tau levels in Cerebrospinal Fluid (CSF) for the overall sample (**A**). Stratified association results by *APOE* for Aβ-42 (**B**), p-tau (**C**), and t-tau (**D**) are presented. Note: A star (*) indicates a significant association (False Discovery Rate < 0.05)

In the replication cohort (Supplementary Table 9 and Fig. 3), PGF2α demonstrated higher CSF levels in A + T + N + compared to A + T-N- (*P* = 1.99 × 10^–2^), similar to the findings from the discovery cohort (Supplementary Table 8). CSF levels of 8, 12-iso-iPF2α VI were significantly higher in A + T + N- compared to A + T-N- patients (*P* = 4.36 × 10^–4^). Additionally, 8-iso-PGF2α (15-F2t-IsoP) showed elevated levels in A + T + N + MCI patients compared to A + T + N- (*P* = 1.17 × 10^–2^), and 5-iPF2a VI levels were increased in A + T + N- compared to A-T-N- categories (*P* = 3.41 × 10^–2^).

### Association of metabolites with ATN biomarkers in *APOE* stratified analysis

To study the role of *APOE* genotype on associations of metabolites in CSF with AD pathology biomarkers, *APOE* stratified analyses were performed (Fig. 3). Of the three metabolites (PGF2α, 5-iPF2α VI, 8,12-iso-iPF2α VI) which showed association with p-tau levels in CSF in the overall analysis (Fig. 3A), PGF2α showed a positive association with p-tau (β = 0.619, *P* = 4.0 × 10^–4^) and t-tau (β = 0.523, *P* = 4.0 × 10^–3^) in only *APOE* ε4 carriers (Fig. 3C-D). Although the heterogeneity p-value was not significant (*APOE* ε4 strata: p-tau = 0.27, t-tau = 0.44), beta values are high in the smaller cohort, indicating the possibility that association was driven by one cohort. The association of 5-iPF2α VI with p-tau (β = 0.308, *P* = 5.0 × 10^–3^) and t-tau (β = 0.288, *P* = 0.011) was significant only in *APOE* ε3 carriers. The isoprostane 8,12-iso-iPF2α VI showed association with p-tau in both *APOE* ε3 (β = 0.293, *P* = 2.0 × 10^–3^) and *APOE* ε4 carriers (β = 0.395, *P* = 3.0 × 10^–3^), while with t-tau in only *APOE* ε3 carriers (*APOE* ε33: β = 0.298, *P* = 3.0 × 10^–3^). 8,12-iso-iPF2α VI showed a negative regression coefficient in association with p-tau and t-tau in *APOE* ε2 carriers. In the association analysis of the metabolite levels as outcome with *APOE* genotypes (Supplementary tables 10), we did not observe altered levels of oxidative stress and inflammatory metabolites between *APOE* ε4 versus *APOE* ε3 and *APOE* ε2 versus *APOE* ε3 as well as *APOE* ε4 versus *APOE* ε2.

### Role of metabolites in MCI to AD dementia progression

The metabolites were also tested for their association with MCI to AD dementia progression in CSF (Supplementary Table 11) and plasma (Supplementary Table 12). The mean follow-up time in AD progressors was 1.42 years (SD = 0.53) and 1.58 years (SD = 0.73) in non-AD progressors. In the ACE cohort, 11 MCI patients also progressed to other types of dementia including vascular dementia (n = 6), semantic dementia (n = 1), Parkinson dementia (n = 1), Lewy Body dementia (n = 2), and Frontotemporal dementia (n = 1). We did not observe significant association of metabolite levels with MCI to AD dementia progression in both models with and without *APOE* adjustment and in *APOE* stratified analysis.

## Discussion

We observed a significant association of isoprostane 8,12-iso-iPF2α VI with increased p-tau and t-tau levels in CSF, while an isoprostane (5-iPF2α VI) and a prostaglandin (PGF2α) showed significant association with only p-tau levels in the overall analysis. In the sensitivity analysis, association of PGF2α with both p-tau and total tau levels, and 8,12-iso-iPF2α VI with p-tau levels was confined to amyloid positive MCI patients. In the *APOE* stratified analysis, PGF2α and 5-iPF2α VI showed significant association with p-tau and t-tau in only *APOE* ε4 and *APOE* ε3 carriers, respectively. Whereas 8,12-iso-iPF2α VI showed association with p-tau and t-tau in both A*POE* ε4 and *APOE* ε3 carriers.

Isoprostanes are the products of lipid peroxidation and established markers of oxidative stress [19]. Our findings are in line with earlier studies reporting the association of an isoprostane 8,12-iso-iPF2α VI with MCI [5] and AD [11] and CSF levels of tau and amyloid in AD patients [10]. The association of 8,12-iso-iPF2α VI with p-tau and t-tau was observed in both *APOE* ε4 and *APOE* ε3 carriers suggesting the oxidative stress in AD is not restricted to *APOE* ε4 carriers. The association of 8,12-iso-iPF2α VI with p-tau levels was observed only in amyloid positive MCI patients. Its increased levels in A + T + N-/ A + T + N + compared with A + T-N-, altogether support its relevance with amyloid induced tau aggregation and oxidative stress. Multiple studies suggest that oxidative stress enhances the phosphorylation [47] through multiple pathways [48], thus enhancing polymerization of tau as neurofibrillary tangles [49]. Future studies are warranted to understand the complex interplay between oxidative stress metabolites (8,12-iso-iPF2α VI), amyloid and tau pathology. The isoprostane 5-iPF2α VI, which is highly correlated with 8,12-iso-iPF2α VI, also showed significantly higher levels in A + T + N + compared with A + T-N- in ACE cohort. Considering the CSF levels of p-tau and t-tau as biomarkers of neurodegeneration and AD progression [50], our findings suggest that isoprostanes (8,12-iso-iPF2α VI, 5-iPF2 α Vi) may increase during the prodromal phase of AD development independent of *APOE* ε4. This is still not clear whether oxidative stress is a cause or consequence, however, higher mean levels of isoprostanes in A-T-N- MCI patients compared to A + T-N- cases, may also suggest the oxidative stress an early event in AD pathology [51] which is exacerbated by amyloid. We also observed increased levels of CSF 8-iso-PGF2α in A + T + N + compared to A + T-N- (*P* = 2.3 × 10^–2^) in the ACE cohort and a similar trend among A + T + N + versus A + T + N- (*P* = 1.2 × 10^–2^) in the replication cohort. This aligns with the reported elevated levels of 8-iso-PGF2α in hippocampal neurons of AD patients, and its weak correlation with p-tau (neurofibrillary tangles) levels during advanced AD pathology compared to controls [52].

The difference in association of specific isoprostanes with AD biomarkers between *APOE* ε3 and *APOE* ε4 carriers is aligned with the relationship of *APOE* isoforms with oxidative stress pathways and lipid peroxidation processes [53]. *APOE* ε3 and *APOE* ε4 alleles may influence oxidative stress in distinct manners, leading to different lipid peroxidation profiles of 5-iPF2α VI and 8,12-iso-iPF2α VI which are two F2 isoprostane regioisomers. The isoprostane 8,12-iso-iPF2α VI was associated with p-tau in both *APOE* ε33 and *APOE* ε4 carriers, but with t-tau only in *APOE* ε33 carriers. This indicates that 8,12-iso-iPF2α VI may be a more general marker of oxidative stress independent of *APOE* ε4. However, the *APOE* ε3 specific association of 8,12-iso-iPF2α VI with t-tau may also suggest nonspecific nature of total-tau marker in reflecting AD specific neurodegeneration.

Among the nine profiled isoprostanes and prostaglandins, only two were detected in plasma samples of ACE cohort. This may be due to low concentrations of these metabolites in plasma or their levels falling below our limit of quantitation. We did not observe any association between plasma levels of isoprostane 8,12-iso-iPF2α VI and 8-iso-PGF2α with CSF levels of Aβ-42, p-tau and t-tau, which might be due to lack of correlation (P = 0.37) between CSF and plasma levels of these specific isoprostanes in our study. This low correlation can be attributed to the low concentration of isoprostanes in plasma along with different clearance mechanisms in blood and CSF. This might also suggest that the origin of the plasma levels of 8,12-iso-iPF2α VI may be different and do not reflect the AD pathology-specific oxidative stress and lipid peroxidation in the brain. Isoprostanes have been shown to associate with AD when measured in CSF, but their plasma levels did not confirm the findings of CSF [54, 55], suggesting that peripheral oxidative stress may not directly reflect oxidative stress status in the central nervous system. Despite a strong positive correlation of plasma and CSF levels of 8-iso-PGF2α, we did not observe its association with ATN biomarkers in overall MCI population in linear regression. This discrepancy underscores the complex relationship between peripheral and central biomarker concentrations in AD research. This relationship can be specific to individual metabolites due to distinct biosynthetic pathways of isoprostanes [56]. Therefore, this highlights the need for further research into the mechanisms underlying these differences.

A prostaglandin PGF2α associates significantly with both p-tau and t-tau in only *APOE* ε4 carriers in overall MCI patients, and in MCI due to AD pathology in non-*APOE* stratified analysis. The *APOE* ε4 not only increases cerebral amyloid pathology, neuroinflammation and tau pathology [57], but also potentiates the impact of amyloid pathology on tau pathology [58]. The association of PGF2α with phosphorylated tau in amyloid positive MCI patients in linear model, along with significantly higher levels in A + T + N + compared with A +T-N- supports the role of PGF2α in neuroinflammation due to amyloid pathology exacerbated tau pathology. PGF2α is one of the most important prostanoids with wide-ranging functions in inflammation, cardiovascular function, and smooth muscle contraction [21, 59]. PGF2α is a product of arachidonic acid metabolism which can be generated through enzymatic mediation by cyclooxygenase-2 (COX-2) and Prostaglandin F Synthase (PGFS) or via autooxidation. In the central nervous system, prostamide/prostaglandin F synthase and cannabinoid receptor 1 (CBR1) both involve the production of PGF2α of which CBR1 is possibly the predominant one [60–62]. Oxidative stress, crucial in AD pathogenesis, has been reported to be associated with increased levels of cytotoxic carbonyl products which consequently induce elevated level of CBR1 enzyme in the brain [63]. Carbonyls from lipid peroxidation modify tau proteins and result in consequent aggregation of phosphorylated tau [64, 65]. Therefore, this may suggest the mechanistic relationship among phosphorylated tau proteins, carbonyl compounds, PGF2α production via CBR1 and oxidative stress in *APOE* ε4 stratified analysis. On the other hand, PGF2α together with F2-series isoprostanes (e.g. 2,3-dinor-8-iso-PGF2α; 5-iPF2α VI; 8,12-iso-iPF2α VI) showed positive associations with p-tau and t-tau in the *APOE* ε4 group (Fig. 3). This may indicate the potentially active contribution of autooxidation pathway mediated PGF2α production. Future mechanistic investigations on which pathway is more actively involved in PGF2α generation should be performed to shed light on the association of prostaglandin/isoprostane generation with *APOE* genotype as well as ATN biomarkers.

In the meta-analysis of overall MCI patients, CSF levels of PGE2 showed associations with Aβ42 which did not pass multiple testing (*FDR* < 0.05), and therefore needs validation. An additional explanation for the weak association of PGE2 and PGA1 with Aβ42, beyond the small sample size, may be the longitudinal stability of Aβ42 compared to p-tau and total tau levels [66]. Nonetheless, CSF PGE2 levels along with four other metabolites (PGF2α, 8,12-iso-iPF2α VI, and 5-iPF2α VI) showed significant associations with CSF Aβ40 levels in the Mannheim/Heidelberg cohort. The positive correlation between CSF levels of PGE2 and Aβ may indicate the role of PGE2 levels in amyloid beta production. This observation is supported by multiple studies that the PGE2 receptors suppresse the neuroprotective effects of microglia, thereby promoting the neuroinflammation [67, 68] and Aβ pathology. This aligns with another study which reported increased levels of PGE2 in AD compared to healthy controls [69]. The ATN category analysis showed that in amyloid positive MCI, the PGE2 levels were lower in A + T + N + compared to A-T-N-, and levels of PGA2 (product of PGE2 dehydration) were significantly lower in A + T + N + compared to A + T-N- in the ACE cohort (Supplementary Table 8). Similar results were reported in a longitudinal study, where CSF PGE2 levels were decreased in AD compared to MCI patients [15]. Early rise in COX-mediated inflammatory response in dementia may explain the initial surge in CSF PGE2 levels, followed by a decline in PGE2 levels due to neuronal loss [15, 70, 71]. Our observations may suggest that the neuroinflammatory role of increased levels of PGE2 may be more relevant before the neurodegeneration stage. Nevertheless, future longitudinal studies are needed to determine whether the positive correlation of CSF levels of PGE2 and Aβ42 is a cause or consequence of amyloid pathology.

One of the major limitations of our study is our limited sample size which challenged our *APOE* stratified analyses. For two study cohorts included in this study, meta-analysis of association of metabolites with AD pathology biomarkers was only available for CSF samples due to the unavailability of plasma samples from Heidelberg/Mannheim memory clinic cohort. Moreover, our study had a short follow-up duration for MCI patients, and the sample size for those progressing from MCI to AD dementia was also limited. Furthermore, recent evidence has revealed the limited prognostic utility of plasma t-tau and has instead proposed the combined additive value by investigating t-tau and neurofilament light (NfL) [72]. In the pursuit of deeper insights into the pathogenesis of AD, future research endeavors should investigate the relationship of oxidative stress/inflammatory metabolites with NfL and neuroimaging phenotypes. Such investigations will provide a more profound understanding of the underlying mechanisms driving Alzheimer's disease pathology. Another limitation is the non-availability of information on Aβ42/ Aβ40 for ACE cohort which is considered a superior marker compared to Aβ42 marker alone.

## Conclusions

In our study, we showed the association of CSF levels of inflammatory (prostaglandins) and oxidative stress (Isoprostanes) related metabolites with biomarkers of AD pathology (Aβ-42, p-tau, t-tau). Robust associations between PGF2α and 8,12-iso-iPF2α VI with tau pathology in amyloid positive participants in both cohorts indicate the role of these metabolites in neuroinflammation and oxidative stress specific to AD pathology. Moreover, our study provides insight into the role of *APOE* in influencing the oxidative stress and inflammatory metabolites during the prodromal phase of AD.

### Supplementary Information

Supplementary Material 1.

Supplementary Material 2.

## Data Availability

The data that support the findings of this study are not openly available due to reasons of sensitivity and are available from the corresponding author upon reasonable request. Data are located in controlled access data storage at Leiden University.

## Abbreviations

AD: Alzheimer’s disease
ATN: Amyloid, Tau, Neurodegeneration
*APOE*: Apolipoprotein E
MCI: Mild cognitive impairment
CSF: Cerebrospinal fluid
p-tau: Phosphorylated tau
t-tau: Total-tau
Aβ: Amyloid-beta
UHPLC–MS/MS: Ultra-high-performance liquid chromatography tandem mass spectrometry
ADAPTED: The Alzheimer’s Disease Apolipoprotein Pathology for Treatment Elucidation and Development consortium
BMI: Body mass index
ACE: Alzheimer Center Barcelona
FDR: False discovery rate
COX-2: Cyclooxygenase-2
PGFS: Prostaglandin F Synthase
CBR1: Cannabinoid receptor 1
NfL: Neurofilament light

## References

[1] Pisoschi AM, Pop A. The role of antioxidants in the chemistry of oxidative stress: a review. Eur J Med Chem. 2015;97:55–74. 25942353 10.1016/j.ejmech.2015.04.040

[2] Uttara B, Singh AV, Zamboni P, Mahajan RT. Oxidative stress and neurodegenerative diseases: a review of upstream and downstream antioxidant therapeutic options. Curr Neuropharmacol. 2009;7:65–74. 19721819 10.2174/157015909787602823 PMC2724665

[3] Radi E, Formichi P, Battisti C, Federico A. Apoptosis and oxidative stress in neurodegenerative diseases. J Alzheimers Dis. 2014;42(Suppl 3):S125–52. 25056458 10.3233/JAD-132738

[4] Pratico D. Oxidative stress hypothesis in Alzheimer’s disease: a reappraisal. Trends Pharmacol Sci. 2008;29:609–15. 18838179 10.1016/j.tips.2008.09.001

[5] Pratico D, et al. Increase of brain oxidative stress in mild cognitive impairment: a possible predictor of Alzheimer disease. Arch Neurol. 2002;59:972–6. 12056933 10.1001/archneur.59.6.972

[6] de Leeuw FA, et al. Blood-based metabolic signatures in Alzheimer’s disease. Alzheimers Dement (Amst). 2017;8:196–207. 28951883 10.1016/j.dadm.2017.07.006 PMC5607205

[7] Abdul-Muneer PM, Chandra N, Haorah J. Interactions of oxidative stress and neurovascular inflammation in the pathogenesis of traumatic brain injury. Mol Neurobiol. 2015;51:966–79. 24865512 10.1007/s12035-014-8752-3 PMC9420084

[8] Heneka MT, et al. Neuroinflammation in Alzheimer’s disease. Lancet Neurol. 2015;14:388–405. 25792098 10.1016/S1474-4422(15)70016-5 PMC5909703

[9] Calsolaro V, Edison P. Neuroinflammation in Alzheimer’s disease: Current evidence and future directions. Alzheimers Dement. 2016;12:719–32. 27179961 10.1016/j.jalz.2016.02.010

[10] Pratico D, et al. Increased 8,12-iso-iPF2alpha-VI in Alzheimer’s disease: correlation of a noninvasive index of lipid peroxidation with disease severity. Ann Neurol. 2000;48:809–12. 11079549 10.1002/1531-8249(200011)48:5<809::AID-ANA19>3.0.CO;2-9

[11] Pratico D, Lee VMY, Trojanowski JQ, Rokach J, Fitzgerald GA. Increased F2-isoprostanes in Alzheimer’s disease: evidence for enhanced lipid peroxidation in vivo. FASEB J. 1998;12:1777–83. 9837868 10.1096/fasebj.12.15.1777

[12] Montine TJ, et al. Increased CSF F2-isoprostane concentration in probable AD. Neurology. 1999;52:562–5. 10025788 10.1212/WNL.52.3.562

[13] Montine TJ, Markesbery WR, Morrow JD, Roberts LJ 2nd. Cerebrospinal fluid F2-isoprostane levels are increased in Alzheimer’s disease. Ann Neurol. 1998;44:410–3. 9749613 10.1002/ana.410440322

[14] Montine TJ, et al. Elevated CSF prostaglandin E2 levels in patients with probable AD. Neurology. 1999;53:1495–8. 10534257 10.1212/WNL.53.7.1495

[15] Combrinck M, et al. Levels of CSF prostaglandin E2, cognitive decline, and survival in Alzheimer’s disease. J Neurol Neurosurg Psychiatry. 2006;77:85–8. 15944180 10.1136/jnnp.2005.063131 PMC2117387

[16] Li G, et al. Cross-sectional and longitudinal relationships between cerebrospinal fluid biomarkers and cognitive function in people without cognitive impairment from across the adult life span. JAMA Neurol. 2014;71:742–51. 24756381 10.1001/jamaneurol.2014.445 PMC4051849

[17] Cummings J. The National Institute on Aging-Alzheimer’s Association Framework on Alzheimer’s disease: Application to clinical trials. Alzheimers Dement. 2019;15:172–8. 29936146 10.1016/j.jalz.2018.05.006 PMC6417790

[18] Rokach J, et al. Total synthesis of isoprostanes: discovery and quantitation in biological systems. Chem Phys Lipids. 2004;128:35–56. 15037151 10.1016/j.chemphyslip.2003.09.011

[19] Pratico D, Lawson JA, Rokach J, FitzGerald GA. The isoprostanes in biology and medicine. Trends Endocrinol Metab. 2001;12:243–7. 11445440 10.1016/S1043-2760(01)00411-8

[20] Hein AM, O’Banion MK. Neuroinflammation and memory: the role of prostaglandins. Mol Neurobiol. 2009;40:15–32. 19365736 10.1007/s12035-009-8066-z PMC3124778

[21] Ricciotti E, FitzGerald GA. Prostaglandins and inflammation. Arterioscler Thromb Vasc Biol. 2011;31:986–1000. 21508345 10.1161/ATVBAHA.110.207449 PMC3081099

[22] Reitz C, Pericak-Vance MA, Foroud T, Mayeux R. A global view of the genetic basis of Alzheimer disease. Nat Rev Neurol. 2023;19:261–77. 37024647 10.1038/s41582-023-00789-z PMC10686263

[23] Lauderback CM, et al. Apolipoprotein E modulates Alzheimer’s Abeta(1–42)-induced oxidative damage to synaptosomes in an allele-specific manner. Brain Res. 2002;924:90–7. 11743999 10.1016/S0006-8993(01)03228-0

[24] Parhizkar S, Holtzman DM. APOE mediated neuroinflammation and neurodegeneration in Alzheimer's disease. Semin Immunol. 2022;59:101594. 10.1016/j.smim.2022.101594. 10.1016/j.smim.2022.101594 PMC9411266 35232622

[25] Boada M, et al. Design of a comprehensive Alzheimer’s disease clinic and research center in Spain to meet critical patient and family needs. Alzheimers Dement. 2014;10:409–15. 24035148 10.1016/j.jalz.2013.03.006 PMC3951687

[26] Petersen RC. Mild cognitive impairment as a diagnostic entity. J Intern Med. 2004;256:183–94. 15324362 10.1111/j.1365-2796.2004.01388.x

[27] Petersen RC, et al. Mild cognitive impairment: clinical characterization and outcome. Arch Neurol. 1999;56:303–8. 10190820 10.1001/archneur.56.3.303

[28] Alegret M, et al. Cut-off scores of a brief neuropsychological battery (NBACE) for Spanish individual adults older than 44 years old. PLoS ONE. 2013;8:e76436. 24146868 10.1371/journal.pone.0076436 PMC3797837

[29] Edition F. Diagnostic and statistical manual of mental disorders. Am Psychiatric Assoc. 2013;21:591–643.

[30] McKhann GM, et al. The diagnosis of dementia due to Alzheimer’s disease: recommendations from the National Institute on Aging-Alzheimer’s Association workgroups on diagnostic guidelines for Alzheimer’s disease. Alzheimers Dement. 2011;7:263–9. 21514250 10.1016/j.jalz.2011.03.005 PMC3312024

[31] Roman GC, et al. Vascular dementia: diagnostic criteria for research studies. Report of the NINDS-AIREN International Workshop. Neurology. 1993;43:250–60. 8094895 10.1212/WNL.43.2.250

[32] Mesulam MM, Grossman M, Hillis A, Kertesz A, Weintraub S. The core and halo of primary progressive aphasia and semantic dementia. Ann Neurol. 2003;54(Suppl 5):S11–4. 12833362 10.1002/ana.10569

[33] McKeith IG, et al. Diagnosis and management of dementia with Lewy bodies: Fourth consensus report of the DLB Consortium. Neurology. 2017;89:88–100. 28592453 10.1212/WNL.0000000000004058 PMC5496518

[34] Vanderstichele H, et al. Standardization of preanalytical aspects of cerebrospinal fluid biomarker testing for Alzheimer’s disease diagnosis: a consensus paper from the Alzheimer’s Biomarkers Standardization Initiative. Alzheimers Dement. 2012;8:65–73. 22047631 10.1016/j.jalz.2011.07.004

[35] Blennow K, Zetterberg H. The application of cerebrospinal fluid biomarkers in early diagnosis of Alzheimer disease. Med Clin North Am. 2013;97:369–76. 23642576 10.1016/j.mcna.2012.12.012

[36] Orellana A, García-González P, Valero S, Montrreal L, de Rojas I, Hernández I, Rosende-Roca M, Vargas L, Tartari JP, Esteban-De Antonio E, Bojaryn U, Narvaiza L, Alarcón-Martín E, Alegret M, Alcolea D, Lleó A, Tárraga L, Pytel V, Cano A, Marquié M, Boada M, Ruiz A. Establishing In-House Cutoffs of CSF Alzheimer's Disease Biomarkers for the AT(N) Stratification of the Alzheimer Center Barcelona Cohort. Int J Mol Sci. 2022;23(13):6891. 10.3390/ijms23136891. 10.3390/ijms23136891 PMC9266894 35805894

[37] Winblad B, et al. Mild cognitive impairment–beyond controversies, towards a consensus: report of the International Working Group on Mild Cognitive Impairment. J Intern Med. 2004;256:240–6. 15324367 10.1111/j.1365-2796.2004.01380.x

[38] Morris JC, et al. The Consortium to Establish a Registry for Alzheimer’s Disease (CERAD). Part I. Clinical and neuropsychological assessment of Alzheimer’s disease. Neurology. 1989;39:1159–65. 2771064 10.1212/WNL.39.9.1159

[39] Walton D. The diagnostic and predictive accuracy of the wechsler memory scale in psychiatric patients over 65. J Ment Sci. 1958;104:1111–8. 13621155 10.1192/bjp.104.437.1111

[40] Reitan RM, Wolfson D. The Trail Making Test as an initial screening procedure for neuropsychological impairment in older children. Arch Clin Neuropsychol. 2004;19:281–8. 15010091 10.1016/S0887-6177(03)00042-8

[41] Kaerst L, et al. Cerebrospinal fluid biomarkers in Alzheimer’s disease, vascular dementia and ischemic stroke patients: a critical analysis. J Neurol. 2013;260:2722–7. 23877436 10.1007/s00415-013-7047-3 PMC3825487

[42] Ahmad S, et al. Association of lysophosphatidic acids with cerebrospinal fluid biomarkers and progression to Alzheimer’s disease. Alzheimers Res Ther. 2020;12:124. 33008436 10.1186/s13195-020-00680-9 PMC7532619

[43] Schoeman JC, et al. Development and application of a UHPLC-MS/MS metabolomics based comprehensive systemic and tissue-specific screening method for inflammatory, oxidative and nitrosative stress. Anal Bioanal Chem. 2018;410:2551–68. 29497765 10.1007/s00216-018-0912-2 PMC5857282

[44] Willer CJ, Li Y, Abecasis GR. METAL: fast and efficient meta-analysis of genomewide association scans. Bioinformatics. 2010;26:2190–1. 20616382 10.1093/bioinformatics/btq340 PMC2922887

[45] Benjamini Y, Hochberg Y. Controlling the false discovery rate: a practical and powerful approach to multiple testing. J Roy Stat Soc: Ser B (Methodol). 1995;57:289–300. 10.1111/j.2517-6161.1995.tb02031.x

[46] van der Lee SJ, et al. The effect of APOE and other common genetic variants on the onset of Alzheimer’s disease and dementia: a community-based cohort study. Lancet Neurol. 2018;17:434–44. 29555425 10.1016/S1474-4422(18)30053-X

[47] Zhu X, et al. Activation of p38 kinase links tau phosphorylation, oxidative stress, and cell cycle-related events in Alzheimer disease. J Neuropathol Exp Neurol. 2000;59:880–8. 11079778 10.1093/jnen/59.10.880

[48] Firdous SM, Khan SA, Maity A. Oxidative stress–mediated neuroinflammation in Alzheimer’s disease. Naunyn-Schmiedeberg's Arch Pharmacol. 2024. 10.1007/s00210-024-03188-3. 10.1007/s00210-024-03188-3 38832985

[49] Gamblin TC, King ME, Kuret J, Berry RW, Binder LI. Oxidative regulation of fatty acid-induced tau polymerization. Biochemistry. 2000;39:14203–10. 11087369 10.1021/bi001876l

[50] Schraen-Maschke S, et al. Tau as a biomarker of neurodegenerative diseases. Biomark Med. 2008;2:363–84. 20477391 10.2217/17520363.2.4.363 PMC2993973

[51] Yao Y, et al. Enhanced brain levels of 8, 12-iso-iPF2α-VI differentiate AD from frontotemporal dementia. Neurology. 2003;61:475–8. 12939420 10.1212/01.WNL.0000070185.02546.5D

[52] Casadesus G, et al. Increased isoprostane and prostaglandin are prominent in neurons in Alzheimer disease. Mol Neurodegener. 2007;2:2. 17241462 10.1186/1750-1326-2-2 PMC1785381

[53] Butterfield DA, Mattson MP. Apolipoprotein E and oxidative stress in brain with relevance to Alzheimer’s disease. Neurobiol Dis. 2020;138:104795. 32036033 10.1016/j.nbd.2020.104795 PMC7085980

[54] Irizarry MC, Yao Y, Hyman BT, Growdon JH, Pratico D. Plasma F2A isoprostane levels in Alzheimer’s and Parkinson’s disease. Neurodegener Dis. 2007;4:403–5. 17934322 10.1159/000107699

[55] Montine TJ, et al. Peripheral F2-isoprostanes and F4-neuroprostanes are not increased in Alzheimer’s disease. Ann Neurol. 2002;52:175–9. 12210787 10.1002/ana.10272

[56] Roberts LJ 2nd, Fessel JP. The biochemistry of the isoprostane, neuroprostane, and isofuran pathways of lipid peroxidation. Chem Phys Lipids. 2004;128:173–86. 15037162 10.1016/j.chemphyslip.2003.09.016

[57] Shi Y, et al. ApoE4 markedly exacerbates tau-mediated neurodegeneration in a mouse model of tauopathy. Nature. 2017;549:523–7. 28959956 10.1038/nature24016 PMC5641217

[58] Therriault J, et al. APOEepsilon4 potentiates the relationship between amyloid-beta and tau pathologies. Mol Psychiatry. 2021;26:5977–88. 32161362 10.1038/s41380-020-0688-6 PMC8758492

[59] Yu Y, et al. Prostaglandin F2alpha elevates blood pressure and promotes atherosclerosis. Proc Natl Acad Sci U S A. 2009;106:7985–90. 19416858 10.1073/pnas.0811834106 PMC2673134

[60] Yoshikawa K, et al. Preferential localization of prostamide/prostaglandin F synthase in myelin sheaths of the central nervous system. Brain Res. 2011;1367:22–32. 20950588 10.1016/j.brainres.2010.10.019

[61] Hayashi H, Fujii Y, Watanabe K, Hayaishi O. Enzymatic formation of prostaglandin F2 alpha in human brain. Neurochem Res. 1990;15:385–92. 2388711 10.1007/BF00969923

[62] Schieber A, Frank RW, Ghisla S. Purification and properties of prostaglandin 9-ketoreductase from pig and human kidney. Identity with human carbonyl reductase. Eur J Biochem. 1992;206:491–502. 1597188 10.1111/j.1432-1033.1992.tb16952.x

[63] Balcz B, Kirchner L, Cairns N, Fountoulakis M, Lubec G. Increased brain protein levels of carbonyl reductase and alcohol dehydrogenase in Down syndrome and Alzheimer's disease. J Neural Transm Suppl. 2001;(61):193-201. 10.1007/978-3-7091-6262-0_15. 10.1007/978-3-7091-6262-0_15 11771743

[64] Liu Q, Raina AK, Smith MA, Sayre LM, Perry G. Hydroxynonenal, toxic carbonyls, and Alzheimer disease. Mol Aspects Med. 2003;24:305–13. 12893008 10.1016/S0098-2997(03)00025-6

[65] Liu Q, et al. Alzheimer-specific epitopes of tau represent lipid peroxidation-induced conformations. Free Radic Biol Med. 2005;38:746–54. 15721985 10.1016/j.freeradbiomed.2004.11.005

[66] Rosenmann H. CSF biomarkers for amyloid and tau pathology in Alzheimer’s disease. J Mol Neurosci. 2012;47:1–14. 22058061 10.1007/s12031-011-9665-5

[67] Liu Q, et al. PGE(2) signaling via the neuronal EP2 receptor increases injury in a model of cerebral ischemia. Proc Natl Acad Sci U S A. 2019;116:10019–24. 31036664 10.1073/pnas.1818544116 PMC6525498

[68] Johansson JU, et al. Prostaglandin signaling suppresses beneficial microglial function in Alzheimer’s disease models. J Clin Invest. 2015;125:350–64. 25485684 10.1172/JCI77487 PMC4382270

[69] Montine T, et al. Elevated CSF prostaglandin E2 levels in patients with probable AD. Neurology. 1999;53:1495. 10534257 10.1212/WNL.53.7.1495

[70] Yermakova AV, O’Banion MK. Downregulation of neuronal cyclooxygenase-2 expression in end stage Alzheimer’s disease. Neurobiol Aging. 2001;22:823–36. 11754989 10.1016/S0197-4580(01)00303-7

[71] Hoozemans JJ, et al. Cyclin D1 and cyclin E are co-localized with cyclo-oxygenase 2 (COX-2) in pyramidal neurons in Alzheimer disease temporal cortex. J Neuropathol Exp Neurol. 2002;61:678–88. 12152783 10.1093/jnen/61.8.678

[72] Marks JD, Syrjanen JA, Graff-Radford J, et al. Comparison of plasma neurofilament light and total tau as neurodegeneration markers: associations with cognitive and neuroimaging outcomes. Alz Res Therapy. 2021;13:199. 10.1186/s13195-021-00944-y. 10.1186/s13195-021-00944-y PMC8672619 34906229

