# Supplementary material for: Association of oxidative stress and inflammatory metabolites with Alzheimer’s disease cerebrospinal fluid biomarkers in mild cognitive impairment
Source: Alzheimers Res Ther. 2024 Jul 30;16:171. doi: 10.1186/s13195-024-01542-4 (PMC11287840; doi:10.1186/s13195-024-01542-4)

**Supplementary figure 1:** Correlation of metabolite levels between plasma and CSF in ACE cohort for 8-iso-PGF2α (A) and 8,12-iso-iPF2α VI (B).


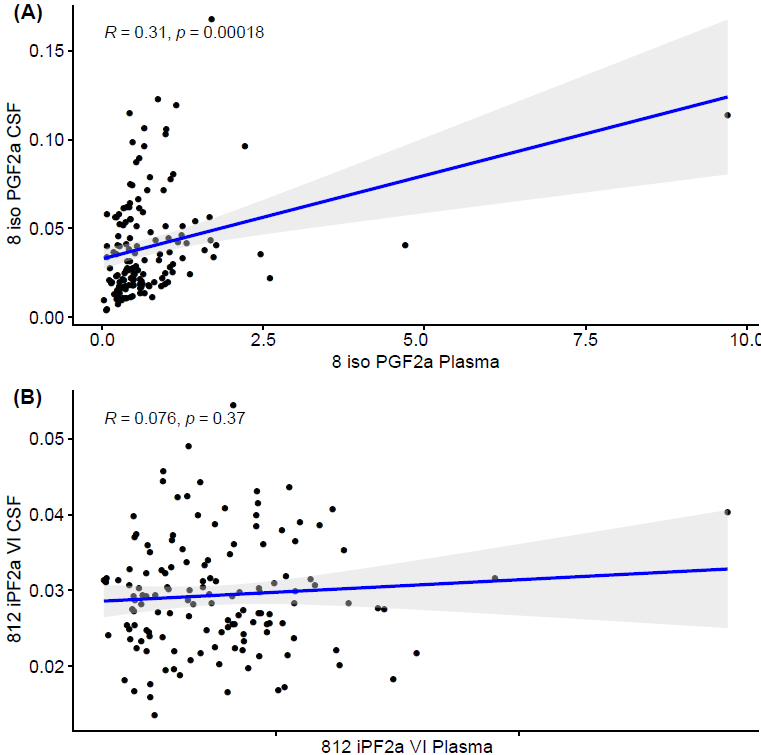


**Supplementary figure 2:** Correlation matrix of metabolites measured in CSF in ACE cohort. Positive correlation is indicated by blue color and red shows a negative correlation between metabolites.


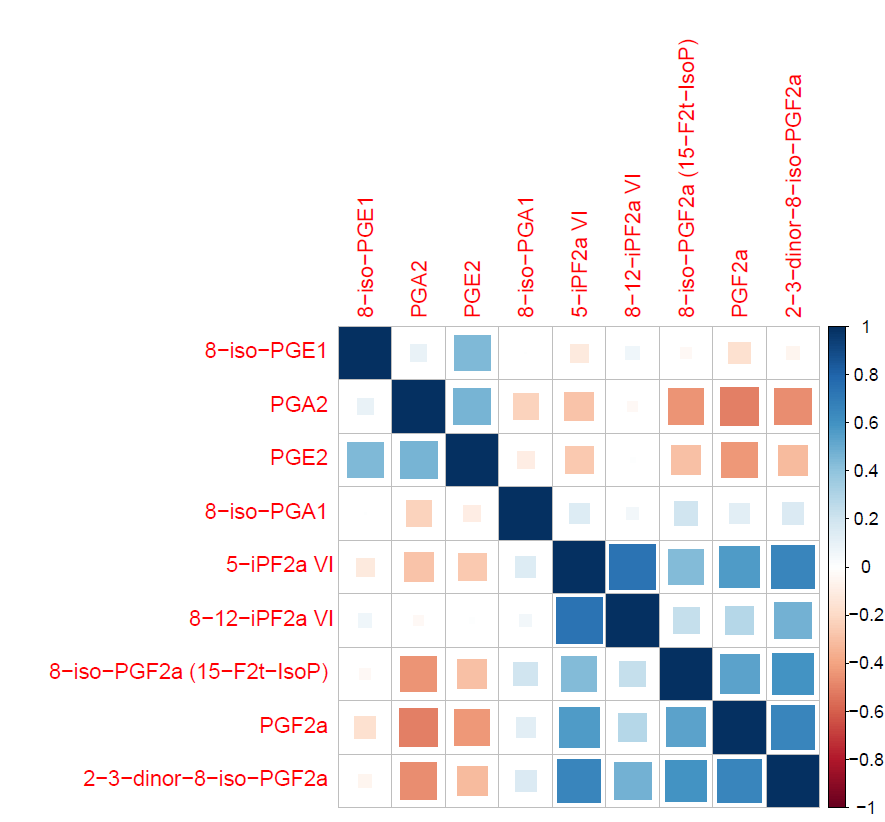


**Supplementary figure 3:** Boxplots illustrating the concentration differences of metabolites across ATN groups in Mannheim/Heidelberg cohort. Each p-value is calculated from a two-tailed t-test assessing the mean differences between groups. P-values are displayed only when they are less than 0.05.


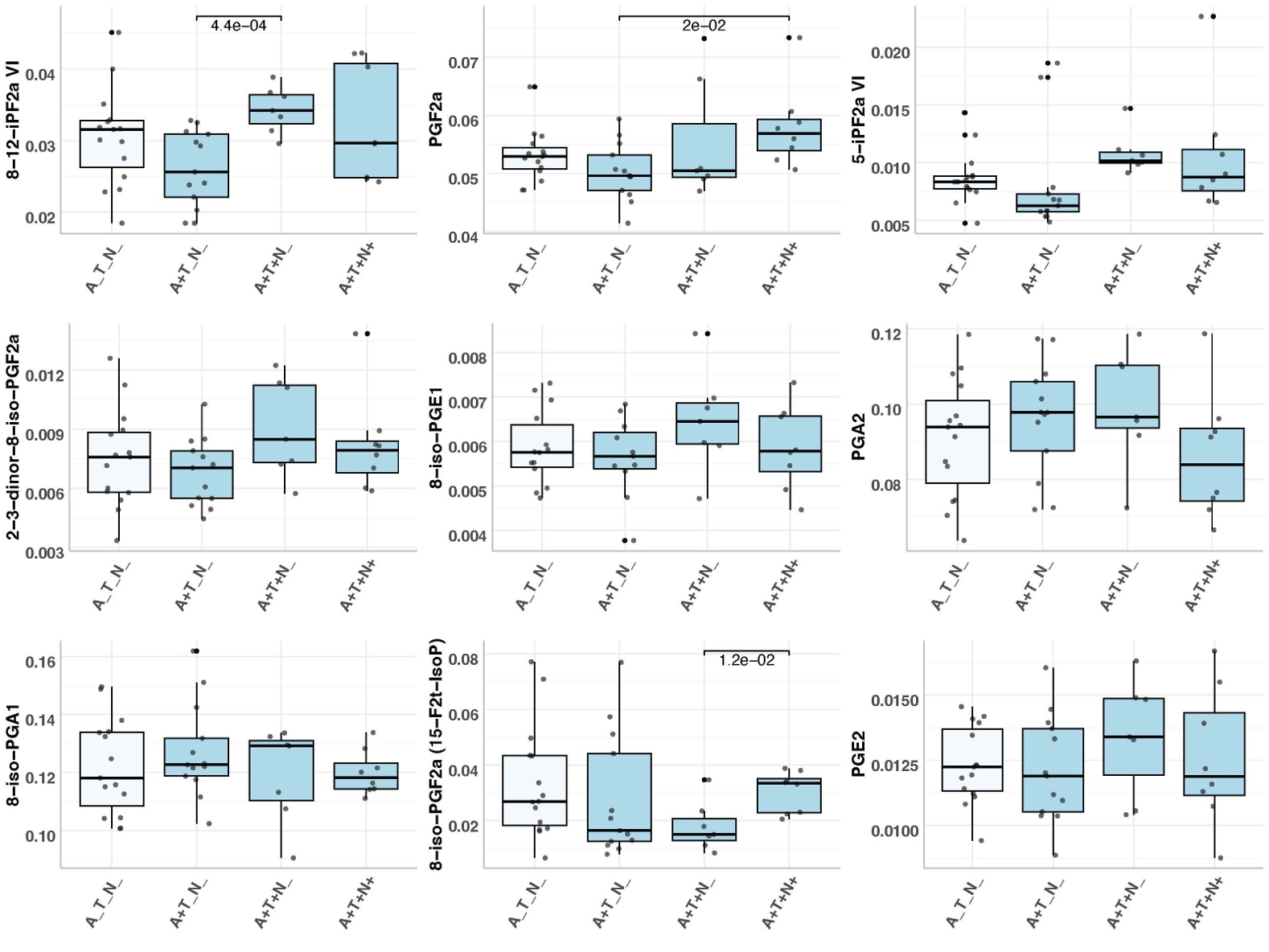

Supplement: Supplementary file 1 — Supplementary Material 1. [file 13195_2024_1542_MOESM1_ESM.docx]
